# Formation and closure of macropinocytic cups in *Dictyostelium*

**DOI:** 10.1016/j.cub.2023.06.017

**Published:** 2023-06-27

**Authors:** Judith E. Lutton, Helena L.E. Coker, Peggy Paschke, Christopher J. Munn, Jason S. King, Till Bretschneider, Robert R. Kay

**Affiliations:** 1 Department of Computer Science, University of Warwick, Coventry CV4 7AL, UK; 2 CAMDU, Warwick Medical School, University of Warwick, Coventry CV4 7AL, UK; 3 MRC Laboratory of Molecular Biology, Cambridge CB2 0QH, UK; 4 School of Biosciences, Western Bank, Sheffield S10 2TN, UK

## Abstract

Macropinocytosis is a conserved endocytic process by which cells engulf droplets of medium into micron-sized vesicles. We use light-sheet microscopy to define an underlying set of principles by which macropinocytic cups are shaped and closed in Dictyostelium amoebae. Cups form around domains of PIP3 stretching almost to their lip and are supported by a specialized F-actin scaffold from lip to base. They are shaped by a ring of actin polymerization created by recruiting Scar/WAVE and Arp2/3 around PIP3 domains, but how cups evolve over time to close and form a vesicle is unknown. Custom 3D analysis shows that PIP3 domains expand from small origins, capturing new membrane into the cup, and crucially, that cups close when domain expansion stalls. We show that cups can close in two ways: either at the lip, by inwardly directed actin polymerization, or the base, by stretching and delamination of the membrane. This provides the basis for a conceptual mechanism whereby closure is brought about by a combination of stalled cup expansion, continued actin polymerization at the lip, and membrane tension. We test this through the use of a biophysical model, which can recapitulate both forms of cup closure and explain how 3D cup structures evolve over time to mediate engulfment.

## Introduction

Macropinocytosis is a large-scale endocytic process where cells engulf droplets of medium into micron-sized vesicles. These vesicles are passaged through the endo-lysosomal system and their contents digested and absorbed.^1–5^ It is conserved across the metazoa, from corals to mammals, and also in the amoebozoa.^6,7^ Despite its discovery nearly 100 years ago^8^ and clear medical importance in cancer cell feeding, immune surveillance, and mRNA vaccine uptake,^9–12^ only recently has macropinocytosis started to attract major attention.^13^ Thus, fundamental questions remain at both the organizational and molecular levels.

Macropinocytosis is driven by actin polymerization beneath the plasma membrane, which in different cells produces cups or sometimes flaps of membrane as the primary macropinocytic structures.^14–16^ A major conceptual challenge is to understand how cups are shaped and close. This requires F-actin to be organized in space over a scale of microns, first to make a cup, and then to bring its lips together for closure, all in the absence of guidance from a solid particle or coat. However the signaling lipid phosphatidylinositol(3,4,5)trisphosphate (PIP3) plays a key, but poorly understood, role in organizing macropinocytosis across species.^14,17–19^

*Dictyostelium* amoebae are prolific macropinocytes, as well as phagocytes, and can imbibe their own volume of medium in a few hours.^20,21^ Macropinosomes form around discrete domains of PIP3 (an ether-linked lipid in *Dictyostelium*^22^) in the plasma membrane, which can be microns across^23,24^ and are genetically essential for macropinocytosis.^18,19^ Domains also include active-Ras, which activates PI3-kinase, and active-Rac, which activates actin polymerization^25^ and are regulated by RasGAPs such as (NeuroFibromatosis 1 (NF1) and RGBARG (RCC1, RacGEF, BAR and RasGEF-containing protein).^26–28^

We have proposed that PIP3 domains are templates for macropinocytic cups,^25^ which they shape by attracting a ring of dendritic actin polymerization to their periphery. This ring is visualized by reporters of the Scar/WAVE complex—an activator of the Arp2/3 complex—which form an irregular necklace around PIP3 domains in all cases investigated. Ringed actin polymerization helps explain how cups are shaped but not necessarily how they close.

Limitations in microscopy have hindered investigation of PIP3 domains and macropinocytic cups. Although cups are readily visible, they are difficult to follow over time and in 3D due to their size, dynamism, and the light sensitivity of cells. It is not known how cups close in *Dictyostelium* or any other cell type, and although we previously obtained static images of Scar/WAVE rings, we could not track them over the lifetime of cups to discover how they participate in expansion and closure.

Light-sheet microscopy gives a new approach to these problems, allowing macropinocytic structures to be followed in 3D from birth to closure.^15,16,25,29^ To match this advance in microscopy, image-analysis methods need extending to 3D time series, to segment cells and PIP3 domains and to measure fluorescent intensities along the plasma membrane.^30^

Here, using lattice light-sheet microscopy and custom computational analysis, we follow macropinocytosis and PIP3 domains in the *Dictyostelium* model with unprecedented detail. We describe how macropinocytic cups are structured and show that they have two distinct ways of closing: at lip or base. We find that PIP3 domains maintain a ring of Scar/ WAVE throughout expansion and closure and that cups close when their PIP3 domain stops expanding. We propose a conceptual model for the evolution of macropinocytic cup shape over time, whereby stalled expansion of domains causes cups to close. This “stalled wave” hypothesis is tested through a simple physical model.

## Results

### Macropinosomes form from cups that can close at lip or base

Using lattice light-sheet microscopy, we followed macropinocytosis in *Dictyostelium* cells for periods of 5–10 min in 3D, at around 0.3 Hz, with two fluorescent channels. Cells were observed at low density in a simple medium, where they maintain macropinocytosis for at least a day.^31^ Using strain Ax2, with paired reporters for PIP3 (PkgE-PH-GFP), marking the membrane of macropinocytic cups,^25^ and F-actin (lifeAct-mCherry) revealing the actin cytoskeleton,^32^ we gained an overview of how macropinosomes form.

**Figure 1:**
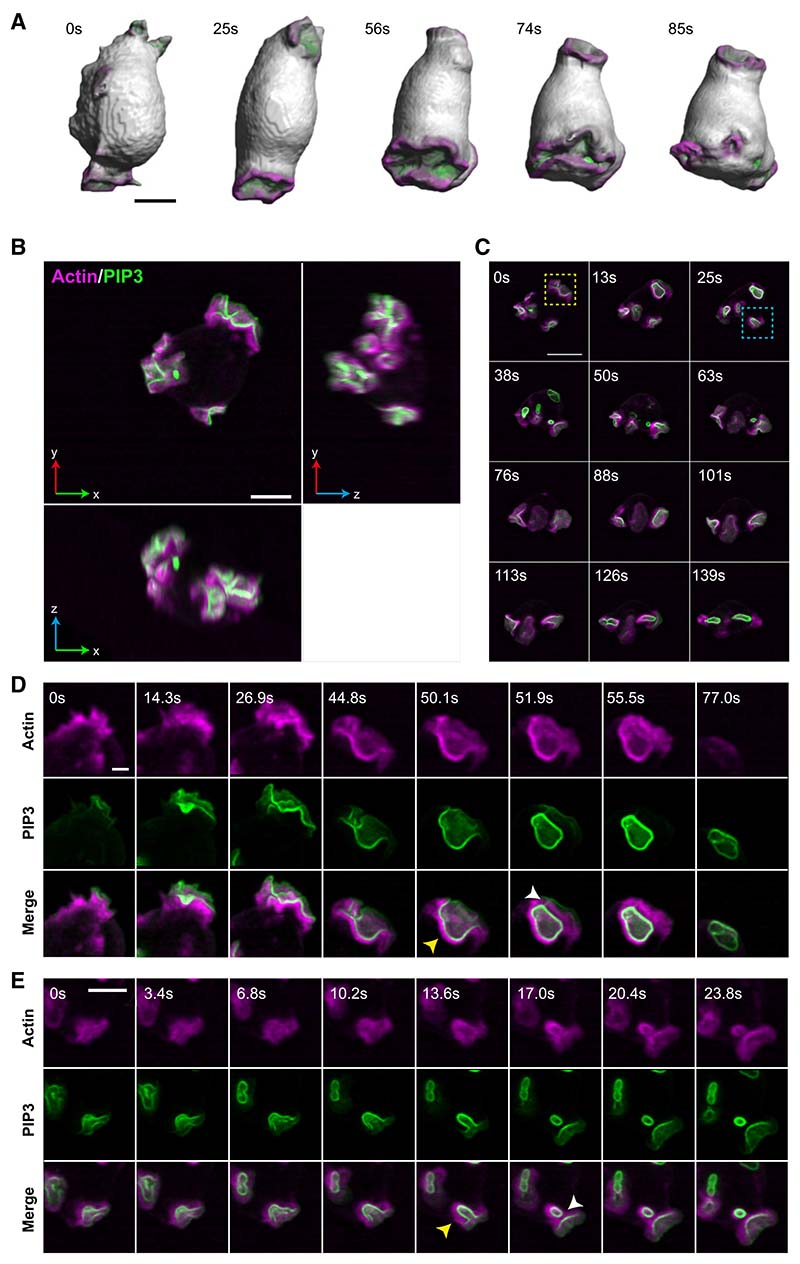
Lattice light-sheet microscopy shows that macropinocytic cups have two ways of closing Images from a lattice light-sheet video of a *Dictyostelium* cell expressing reporters for PIP3 (green) and F-actin (magenta). The PIP3 reporter reveals the PIP3 domain of macropinocytic cups and early macropinosomes and the F-actin reporter reveals their structural scaffold. (A) Surface rendered images of cup formation to show cell shape changes in 3D, see Video S1 for complete series and corresponding fluorescence images. (B) Orthogonal projections of a single cell (x, y top left) having three macropinocytic areas. (C) Time sequence from the same cell. Yellow box: cup closing at the lip releases a large macropinosome at 13 s, whose PIP3 signal intensifies and then fades away by 50 s. The domain itself is extinguished. Blue box: successive basal closures at a second PIP3 domain. Small macropinosomes are released at 38 and 63 s, before the domain closes at the lip at 139 s and is extinguished. (D) Lip closure at higher temporal resolution with split colors, corresponding to the yellow box. Yellow arrow at 50.1 s indicated the F-actin scaffold supporting the cup; at 51.9 s a tether between macropinosome and the cell surface is seen with the PIP3 (white arrow). (E) Basal closure at higher temporal resolution with split colors, corresponding to the blue box. Arrow at 13.6 s: F-actin scaffold; at 17.0 s: newly separated macropinosome and persistent PIP3 in plasma membrane. Ax2 cells transformed with pPI304 to express reporters for PIP3 (PkgE-PH-GFP) and F-actin (lifeAct-mCherry) were viewed in imaging medium by LLSM with full volumes taken every 1.7 s. Maximum intensity projections, except for (A). Scale bars: 5 μm in (A) and (B) and 2 μm in (C) and (D). See Videos S1, S2, and S3 for full sequences. See Figure S1 and Video S4 for examples of complex cup closure, and cups in non-axenic cells, and Figure S2 for cup formation in Ras/PIP3 signaling mutants.

Surface rendering of cells shows a series of cupped, crater-like projections on their surface (Figure 1; Video S1), similar to the “crowns” of the actin-binding protein coronin described in earlier work.^20,33^ These cups expand and close, with maximum-intensity projections showing macropinosomes sealing and being released from the plasma membrane (Video S2). The released macropinosomes retain an F-actin coat and high levels of PIP3 in their membrane but rapidly become invisible as these are lost.

Cups form on any exposed part of cells and frequently move over the surface as they develop. On average, cells have 2.71 ± 1.71 PIP3 domains at any moment and produce 1.8 ± 1.2 macropinosomes per min (Table 1), ranging up to more than 5 μm in their longest dimension. Rates and sizes vary greatly. More rarely, cells produce planar PIP3 domains on their base (“basal waves”^34,35^), which do not produce macropinosomes unless they escape up the side of the cell. We regard these as frustrated macropinosomes.

Overall, *Dictyostelium* cells resemble macrophages in forming macropinosomes from cups,^14^ but differ in that we do not observe them forming from linear ruffles or flaps, nor is there a major involvement of F-actin “tentpoles.”^15,16^

### Macropinocytic cups can close in two different ways

Unexpectedly, we found that cups can close in two different ways—lip and basal closures—of which lip closure is roughly twice as common (Figure 1; Table 1; Videos S2 and S3). In lip closure, cups close at or near the lip and include nearly all the PIP3 domain in the resulting macropinosome. Residual PIP3 in the plasma membrane fades away, extinguishing the domain and terminating macropinocytosis at that site. In a complex variant, macropinocytic cups form lobes, which appear as overlapping arcs of PIP3-reporter in maximum-intensity projections. When these composite structures close, several macropinosomes are released almost simultaneously (Figures S1A and S1B; Video S4).

In basal closure, cups deepen and narrow, then constrict locally before pinching off a macropinosome near the base. A large proportion of the PIP3 domain remains in the plasma membrane, where it can yield further macropinosomes. This sequence generally terminates by closure at the lip and extinction of the PIP3 domain.

Macropinosomes formed by either route frequently remain attached to the cell surface by a thin tether, which can persist for 10 s or more, before rupturing (Figure 1C, 51.9 s; also Figures S1E and S1G). Because tethers are visible with the PIP3 reporter, we assume that they are narrow tubes of membrane, although the individual walls are not resolved. This implies that macropinosomes are not fully sealed and released until the tether is ruptured—a phenomenon that is under further investigation.

Cups can also fail. Most commonly, the PIP3 domain simply fades away, followed by its associated F-actin. Or there can be an abortive closure in which the cup constricts but does not close (Figure S1C; Video S4).

### Macropinocytosis is similar in non-axenic cells

To test the generality of our observations, we examined macropinocytosis in strain DdB, which is an early derivative of the original *D. discoideum* isolate NC4, and parent to Ax2.^36^ Ax2 was selected from DdB for growth in standard liquid medium, which is largely due to deletion of the RasGAP NF1, resulting in Ras activation and massively upregulated macropinocytosis.^26^

DdB cells (with intact NF1) were grown in enriched liquid medium to stimulate macropinocytosis and examined using the PIP3/F-actin reporter combination. They produce generally smaller PIP3 domains and macropinosomes than Ax2 cells. Both lip and basal closures, as well as tethers are observed, showing that these are common features of *Dictyostelium* macropinocytosis (Figures S1D–S1G; Video S4).

### The structure of macropinocytic cups

**Figure 2:**
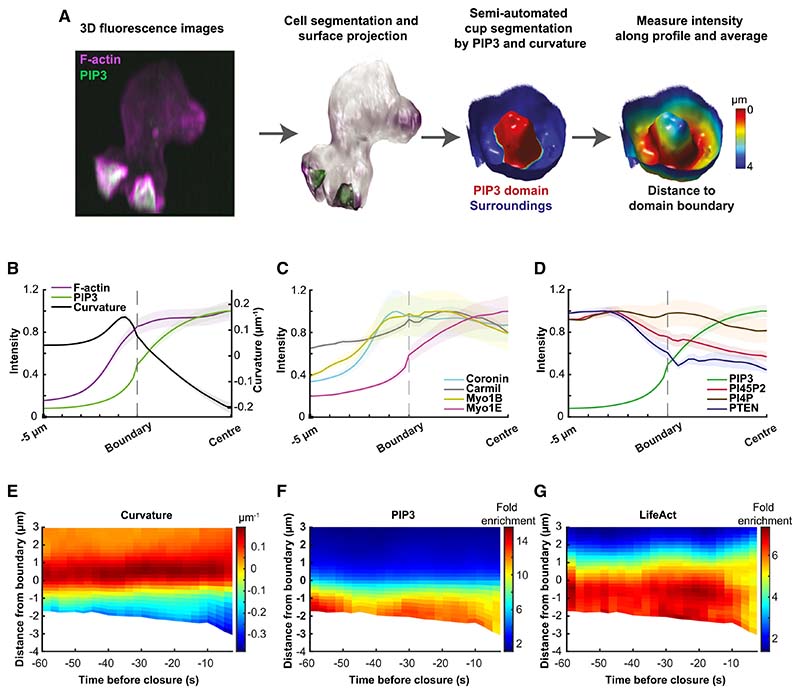
Macropinocytic cups are organized around PIP3 domains, supported by specialized F-actin scaffolds and produce phosphoinosi-tide gradients (A) Mapping strategy: PIP3 domains were defined in a two-step process, yielding first the cell surface segmented by a 3D method using both fluorescent reporters; and then the PIP3 domains, segmented using only the PIP3 reporter, and including a curvature correction for spill-over across membrane folds. Domains were inspected and annotated manually using virtual reality. Vesicles not attached to the plasma membrane are excluded by this process. The lip of cups is defined by the inflection in membrane curvature. (B–D) Average distribution of reporters in macropinocytic cups. Profiles are aligned to the PIP3 domain boundary (“Boundary”) with distances to the center of the domain (“Center”) normalized, whereas distances beyond the boundary are real; shaded areas show standard error. The number of cups analyzed is given in brackets. (B) PIP3 domain (n = 181) and F-actin (n = 48) scaffold: the PIP3 domain occupies the inner surface of the cup, with some spill-over beyond the lip, and the cup is supported by a continuous F-actin scaffold from bottom to beyond the lip. (C) Cytoskeletal proteins: coronin (n = 11), carmil (n = 11) (modestly), myosin1B (n = 7), and myosin1E (n = 12) are all concentrated in macropinocytic cups, with the PIP3-binding myosin1E mirroring the PIP3 distribution and coronin concentrated just beyond the lip. (D) Phosphoinositide gradients: PIP3 is strongly graded with its peak toward the center of the domain, whereas PTEN (n = 6), which breaks PIP3 down, is excluded from PIP3 domains. PI4,5P2 (n = 7), the substrate for PIP3 production, shows an outside-in gradient. This is consistent with PIP3 being produced within PIP3 domains and diffusing out for destruction by PTEN; whereas PI4,5P is depleted within the domain by PIP3 formation and replenished by diffusion from outside. PI4P (n = 7) shows little variation. (E–G) Space-time plots over the last minute before macropinocytic cups close. Both lip and basal closures are included. Cups deepen particularly in the last 10 s before closure, as shown by the increasingly negative distance from the boundary. There is evidence for increased accumulation of PIP3 (n = 21) and F-actin (n = 16) at 10–40 s before closure. All data were obtained using Ax2 cells expressing a PIP3 reporter paired with the reporter of interest. All error bars represent standard error. Example images of each reporter are shown in Figure S3.

To understand how macropinocytic cups are structured and how they change during closure, we devised computational tools to map their individual components over space and time and to combine these into composite maps. For microscopy, a reporter of interest was combined with a constant PIP3 reporter and domains defined by first segmenting the cell surface using both reporters and then the domain itself, using only the PIP3 reporter (Figure 2A). Intensities of a panel of reporters (see STAR Methods) were measured along the membrane from the domain boundary, with the bottom of the cup providing a second reference point, and geometric parameters calculated, including domain area and membrane curvature. This provides the first comprehensive 3D map compiling lipid signaling molecules, cytoskeletal regulators, and geometric features over time.

### PIP3 domains occupy the inner face of macropinocytic cups and are essential for macropinocytosis

We first asked where PIP3 domains are located with respect to the lip of the cup, which was defined by the inflection in membrane curvature. The domain boundary (defined by Otsu thresholding^37^) falls just inside the lip, with PIP3 levels elevated over the entire inner surface of the cup but falling off beyond the lip (Figures 2B, 2E, and 2F). PIP3 also shows a striking gradient within domains, with its high point at their base.

Because PIP3 domains are central to our model, we further tested their importance by examining mutants that either abolish or expand them.^19,25^ All have moderate or severe defects in fluid uptake. At one extreme, in a mutant lacking all five “type-1” PI3-kinases,^38^ PIP3 domains are abolished, but the underlying domains of active Ras still form, although appearing somewhat diffuse. These are incapable of forming macropinosomes (Figure S2; Table 1). Conversely, when phosphatase and tensin homologue (PTEN) is deleted, the PIP3 domains grow to encompass the entire cell surface but are capable of only very inefficient macropinocytosis, forming occasional small macropinosomes. A similar but less extreme phenotype is produced by deleting the RasGAP, RGBARG.^27^ Thus, PIP3 domains are essential, or nearly so, for macropinocytosis, but they must be properly regulated.

PI4,5P2—the substrate for synthesis of PIP3 by PI3-kinases—has a reciprocal distribution to PIP3, with lower levels inside macropinocytic cups than outside (Figure 2D). PTEN, which reverts PIP3 to PI4,5P2, also has a reciprocal distribution, with high levels outside the cup and over the whole plasma membrane, and low levels within the cup, consistent with previous reports.^39^

PI4P, a relatively abundant phosphoinositide of the plasma membrane, shows little change in distribution in PIP3 domains, whereas PI3,4P2 is near background in macropinocytic cups but increases strongly once they close (not shown).

### Macropinocytic cups are mechanically supported by an F-actin scaffold

The flexible plasma membrane requires mechanical support to shape it into a cup. To understand how this is achieved, we mapped F-actin and some of its binding proteins in cups. F-actin forms a continuous scaffold, extending from top to bottom of cups and some distance beyond the rim (Figures 2B and 2G; see also Figures 1B and 1C; Videos S2, S3, and S4). This is most obvious in deep cups just before closure, and it is also clear that the scaffold is thicker in the cup than in more remote areas of the plasma membrane, consistent with it providing structural support.

F-actin-binding proteins are recruited in distinct spatial patterns, broadly confirming previous work (Figures 2C and S3). Coronin has a preference for newly formed filaments and regulates actin dynamics.^33,40^ It is strongly recruited to cups, concentrating toward the lip. Single-headed myosins link F-actin to membranes,^41^ with several strongly recruited to macropinocytic cups. These localize differentially, with myosin-1B recruited toward the periphery of cups, where its staining is often fragmented, whereas the PIP3-binding myosin-1E is evenly concentrated toward the base, closely matching the distribution of PIP3, as previously described.^42,43^ The abundance of myosin-1 proteins in cups suggests that the plasma membrane is more firmly attached to the actin cytoskeleton in cups than elsewhere. Carmil^44^ is only modestly enriched in cups.

Although microtubules (labeled with GFP-alpha-tubulin) moved actively within the cytoplasm and make fleeting contacts with macropinocytic structures, we did not detect any prolonged association between them suggestive of a structural role in cups (not shown). We did not notice any appreciable differences between cups that close at lip or rim, and both were included in the analysis. Nor were any acute changes detected in the patterns of any of the reporters found before cups closed, except possibly in F-actin, whose signal intensified about 20 s before closure as shown by space-time plots (Figures 2E–2G).

### PIP3 domains activate a ring of dendritic actin polymerization around themselves

**Figure 3:**
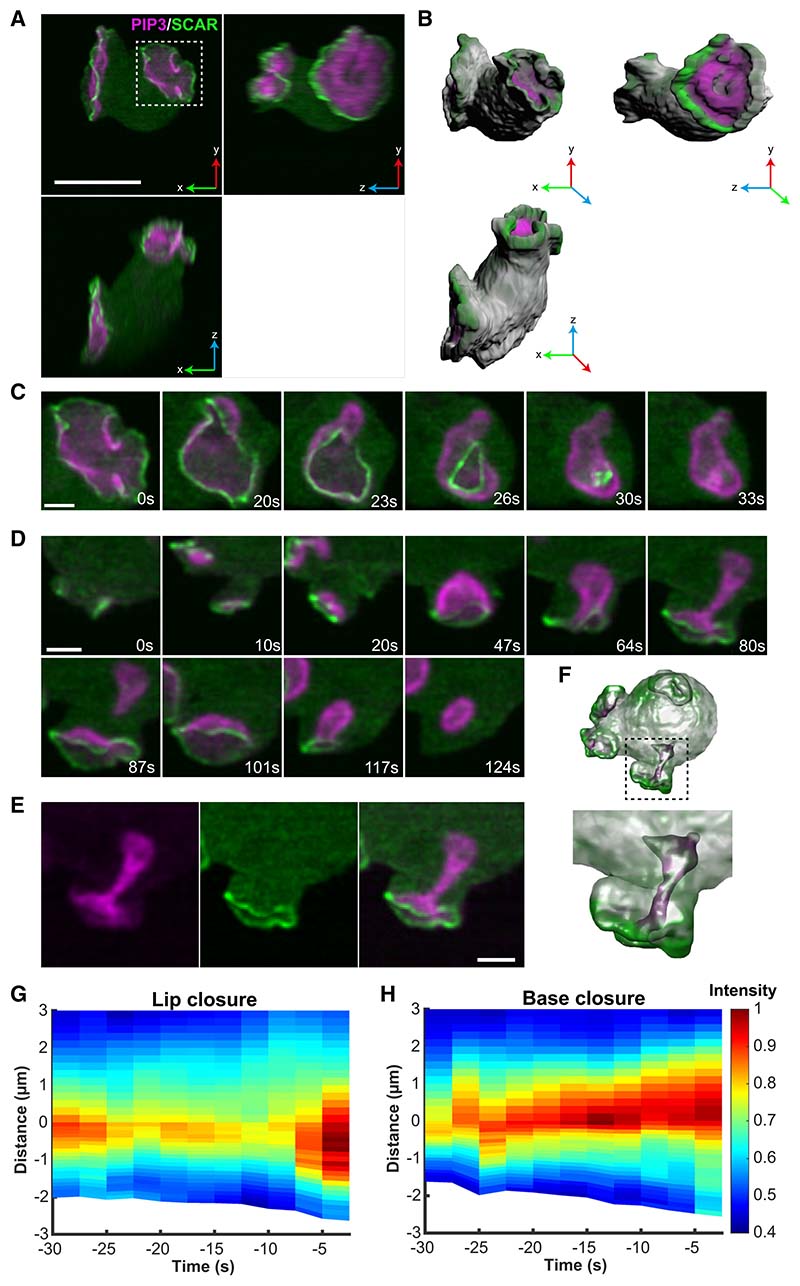
PIP3 domains shape cupped structures by attracting a ring of dendritic actin polymerization to their periphery Dendritic actin polymerization is initiated by the Arp2/3 complex, which is activated by the Scar/WAVE complex. (A) Orthogonal maximum intensity projections of a single Ax2 cell with two macropinocytic cups, expressing reporters for PIP3 (magenta) and the NAP subunit of the Scar/WAVE complex (green). (B) A 3D surface rendering of the frame shown in (A). (C) En face view of a cup closing at the lip, showing a contracting ring of Scar/WAVE, indicating that closure is driven by actin polymerization at the lip. (D) Transverse view of a cup producing macropinosomes by basal closure (87 s) and lip closure (124 s). Scar/WAVE is present at the lip of the cup throughout, but not at the site of the basal closure. Lip closure extinguishes the domain. (E) Split channels of the 80 s panel of (D). (F) Semi-transparent 3D rendering of the basal closure in (E), to show the invaginating tube. (G and H) Space/time plots of the distribution of Scar/WAVE in cups closing either at the lip (n = 7) or base (n = 6) showing the continuous presence of Scar/WAVE at or near the lip of the cup. Closure occurs at 0 s. The edge of the PIP3 domain is set at zero, with negative numbers representing distances inside the cup. Scale bars: 5 μm in (A) and (B) and 2 μm in (C) and (D). See Video S5 for full sequence of Scar/WAVE and Video S6 for Arp2/3 localization.

To understand how the scaffold of cups is shaped, we mapped the sites of dendritic actin polymerization, using recruitment of the Scar/WAVE complex—which activates dendritic actin polymerization—as a proxy. Using a tagged NAP (Nck-associated protein) subunit instead of HSPC300 as used previously,^25^ we again found that Scar/WAVE forms striking rings around PIP3 domains, which could now be followed through the entire lifetime of cups (Figure 3; Video S5). This showed that Scar/ WAVE is recruited around PIP3 domains from their earliest origin through to closure. The persistence of Scar/WAVE at the edge of PIP3 domains is confirmed by space-time plots (Figures 3F and 3G). A reporter for the Arp2/3 complex, which is activated by Scar/WAVE, is recruited in a similar manner (Figure S3B; Video S6), as also recently reported.^45^

These results show that macropinocytic cups are organized around PIP3 domains, which continuously activate a ring of actin polymerization at their periphery. This creates a cup-shaped F-actin scaffold, including specialist F-actin-binding proteins. In the concave parts of the cup, the membrane must be linked to the scaffold strongly enough to prevent it from detaching (delaminating). Myosin-1, and other cross-linking proteins, may perform this role. The general structure of the cup does not appear to change as it switches from expansion to closure, implying a biophysical rather than molecular mechanism for closure.

### How cups close

We examined cups closing at the lip in en face views, using the Scar/WAVE reporter. This clearly shows that these cups close by concerted inward movement of the lips, until the orifice is sealed (Figure 3C; Video S5). We rarely, if ever, observed flaps of membrane sealing them, and although F-actin tentpoles sometimes formed, we did not observe them twisting to seal the cup.^15^

Closure at the lip could be caused by a purse string, whose constriction is driven by myosin-II contraction, as proposed for phagocytic cups.^46^ However, we could not detect myosin-II at the lip, or elsewhere in cups (Figure S3) and macropinocytosis is insensitive to blebbistatin inhibition.^31,47^ Instead, the Scar/ WAVE reporter is strongly recruited to the lip throughout closure, indicating that actin polymerization continues there. We therefore propose that cups close at the lip by continued actin polymerization, which becomes redirected inward.

Examining basal closures in a similar way, we found that Scar/WAVE is not detectably recruited at the site of closure. This is shown in Figure 3D (Video S5), where a basal closure is followed by a lip closure. Scar/WAVE is not recruited to the basal closure site but is to the succeeding lip closure, providing a positive control for the reporter. This indicates that base closure is not driven by localized actin polymerization at the site of closure.

Significantly, Scar/WAVE remains at the lip of cups during closure at the base, indicating that actin polymerization continues at the lip but does not turn inward; instead, it may cause closure indirectly. By analogy with lamellipodia, actin polymerization at the lip of a cup should create a “retrograde flow” of F-actin away from the lip. This is expected to deepen the cup, driving it into the cell as is observed (see Figure S4). Assuming retrograde flow applies force to the membrane, then this will be stretched, increasing tension until eventually a macropinosome is pinched off, giving a basal closure.

**Figure 4:**
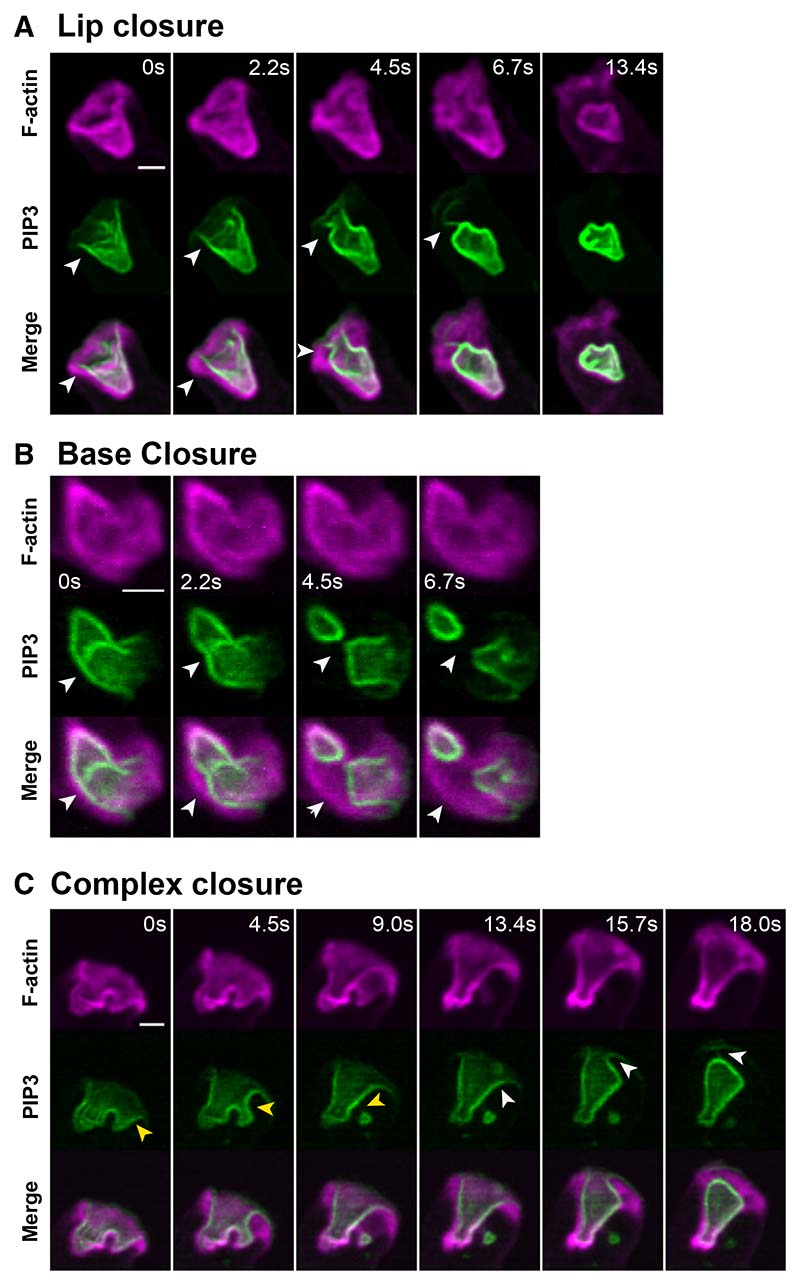
Delamination during cup closure: evidence that the membrane within cups is under tension By delamination we mean the separation of the plasma membrane from the underlying actin cytoskeleton and take this as evidence that the membrane is under tension. Delamination most usually occurs as a macropinosome is sealing. (A) Closure just below the lip with delamination arrowed. Also, note the tether at 6.7 s (arrowed). (B) Base closure with delamination arrowed. (C) Complex closure with delamination arrowed. Arrowheads indicate the points at which delamination occurs, with different colors indicating different events. Ax2 cells expressing reporters for PIP3 and F-actin. Scale bars: 2 μm. Full image sequences shown in Video S7.

Detailed observation of basal closure shows that the PIP3-positive membrane often delaminates from the F-actin scaffold at the site of closure (Figure 4; Video S7). This confirms both that the membrane is under significant tension and that local pinching by the actin cytoskeleton is not the cause of closure. Similar delaminations are also observed with cups closing near the lip, indicating that membrane tension also contributes to lip closure.

Superficially, the two closure methods appear to have different mechanisms, but both involve continued actin polymerization at the lip, driven by Scar/WAVE. We propose that cups close at the lip by redirecting this actin polymerization inward and at the base by using it to deepen the cup, increasing membrane tension until delamination and basal sealing of a macropinosome occurs.

### Macropinocytic cups close when their PIP3 domain ceases expanding

What triggers macropinocytic cup closure? We noticed that cups tend to close when their PIP3 domain stops expanding. Because this leads to a plausible model for closure (next section), we made detailed measurements of domain area to test the correlation.

**Figure 5:**
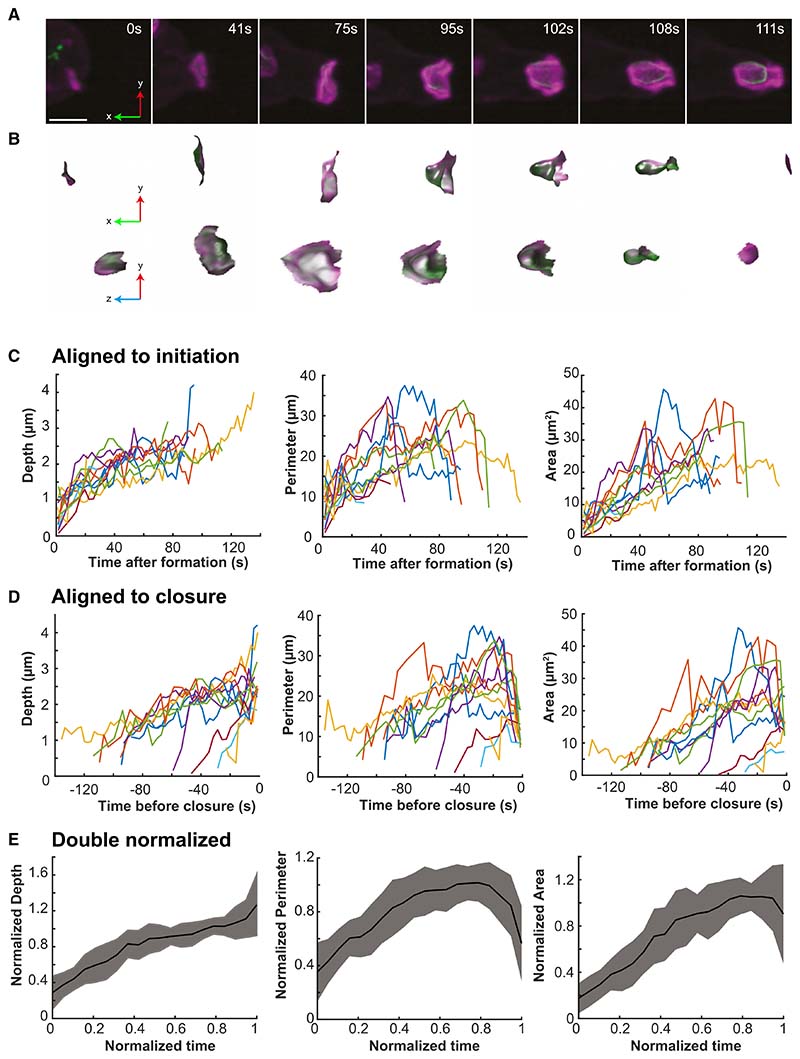
Macropinocytic cups close when their PIP3 domain stops expanding A set of 11 cups that closed at the lip and could be followed from the first appearance of their PIP3 domain was analyzed. (A) Representative domain, which originated in association with a small patch of F-actin, then expanded, deepened, and closed at the lip to yield a macropinosome after 111 s. (B) PIP3 domains corresponding to (A) dissected out by the double segmentation procedure outlined in Figure 2. Shown enlarged in transverse and en face views. The macropinosome in the last frame is not visible because it is no longer attached to the cell surface. (C and D) Depth, perimeter, and area of individual domains followed from origin to closure and aligned either by time of origin or of closure. Although trends can be discerned, there is considerable variability, especially in lifetime, which varies from 30 to 130 s. (E) Double normalized plots of domain depth, perimeter, and area. Strikingly, domains stop expanding in area before they close. The lifetime for each domain is normalized to 1 and the dependent variable to the 75^th^ centile. Error bars represent standard error. The time of closure (0 s) is taken as the last frame in which the macropinocytic cup is unclosed. Ax2 cells expressing reporters for PIP3 (PkgE-PH-GFP) and F-actin (lifeAct-mCherry). Scale bars: 2 μm. See Video S8. Comparison of lip and basal closures are shown in Figure S4.

First, we analyzed a set of simple domains that did not split and could be followed from their origin to their closure at the lip (Figures 5A and 5B; Video S8). All originate with a small PIP3 domain associated with a pre-existing focus of F-actin (11/11 cases). This F-actin focus is usually small, but occasionally can be a pseudopod, which is then subverted into a macropinocytic cup. The domains were dissected out computationally at each time point and their area, perimeter, and depth measured. Plots are strikingly variable, whether aligned by domain initiation or closure (Figures 5C and 5D) primarily due to their spread of size and lifetime (30–130 s). To better discern overall trends, we combined plots by normalizing both time and the geometric parameters (Figure 5E).

The life history of an average domain can be divided into phases. In the first, it expands with a steadily increasing area, while deepening and increasing in perimeter. We measured the rate of area increase in the expansion phase, both in the selected set of domains and in a larger set, obtaining values of 0.35 ± 0.16 μm^2^ s^−1^ (n = 11) and 0.30 ± 0.34 μm^2^ s^−1^ (n = 27), respectively (Table 1). Because the area increases approximately linearly with time, the domain boundary moves at a decreasing speed (proportional to 1/r for a circular domain). This can be regarded as a phase where an expanding wave of actin polymerization captures membrane into the cup, which remains relatively shallow and open. In the second phase, starting at around 0.6 of normalized lifetime, the domain slows and stops expanding.

Finally, the perimeter decreases toward zero as the cup closes, the depth may also increase, whereas the area holds steady or decreases slightly. These last two phases can be regarded as a period in which a nearly constant area of membrane is manipulated by the actin cytoskeleton into a closed vesicle.

In a second test of the correlation, we selected cups which could be followed for the last minute before closure and classified them as either lip or basal closures (Figure S4). In lip closures, the area of the domain does not increase in this period and may even decrease just before closure. Cup depth also only increases slightly, whereas perimeter decreases rapidly in the last 20 s, confirming our previous observations. Basal closure differs: although the area also does not increase over the last 30 s, the cup deepens around 2-fold before a macropinosome is pinched off.

These different approaches show that macropinocytic cups close when their PIP3 domain stop expanding. This suggests a mechanistic hypothesis: stalled expansion of their PIP3 domain causes macropinocytic cups to close.

### A stalled wave model for macropinocytic cup closure

To formalize our proposed mechanism and test its feasibility, we built a simple model based on the following propositions derived from our observations. (1) Dendritic actin polymerization occurs in a ring around the perimeter of PIP3 domains. It is protrusive and pushes against the membrane. (2) Macropinocytic cups are supported by a continuous F-actin scaffold. (3)The plasma membrane adheres to this scaffold via cross-linking molecules, such as myosin-1, and therefore resists delamination. (4) Cups close when expansion of the PIP3 domain stalls.

**Figure 6:**
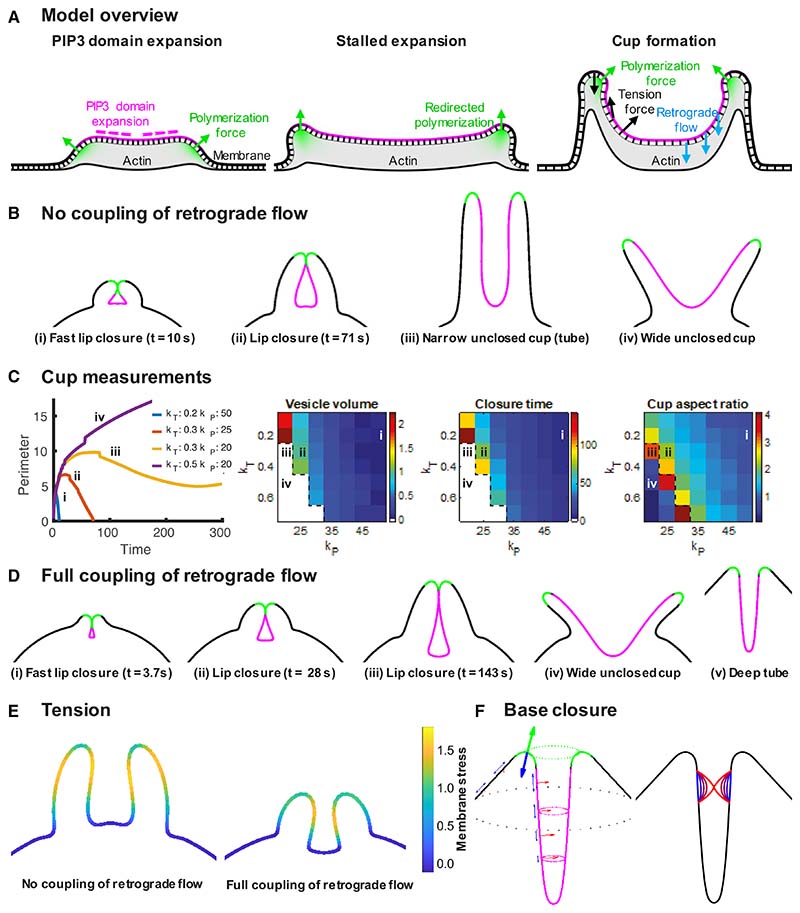
Stalled wave model for macropinocytic cup closure Macropinocytic cups are formed by PIP3 domains (magenta) that are encircled by a ring of protrusive actin polymerization (green). See Video S7. (A) Overview of the model: PIP3 domains first expand, capturing membrane into the cup as the wave of actin polymerization moves outward; then slow and stall with the actin polymerization now remaining under the same area of membrane. The cup deepens and closes either at the lip or base depending on whether the zone of actin polymerization turns inward or not. Actin polymerization under the membrane causes an outward force (green arrows) and an opposite reaction force that can be transmitted to the membrane of the cup by retrograde flow of F-actin (blue arrows); the tension force creates a force whose direction depends on curvature and the elastic tension gradient (black arrows). (B) Outcomes of the model when the force from retrograde flow is not applied to the membrane of the cup. Lip closures, tube-like cups, which potentially close at the base and wide, unclosed cups are produced, depending on the input parameters. (C) Membrane tension and actin protrusive forces control the type of closure (k_T_, membrane tension force coefficient; k_P_, actin polymerization force coefficient). The domain perimeter, vesicle volume, closure time, and aspect ratio of the cups at closure are shown for different combinations of tension and protrusive forces; Roman numerals refer to the cups shown in (B). (D) Outcomes of the model when the force from retrograde flow is fully applied to the membrane of the cup, shown with the same parameters as (B) for (i)–(iv) and k_T_ = 20; k_P_ =100 for (v). Transmission of this force to the membrane increases the likelihood of cups closing and at high elastic moduli, can cause cups to penetrate deeply into the cell. (E) Tension gradients created in the cup. (F) Closure at the base produced by the inward component of membrane tension. This is modeled as a 3D process, which acts to minimize the surface area of the tube. Given a sufficiently large delaminated region, this process leads to closure. Lines represent the membrane at different time points, from blue (earlier) to red (later). Full timeseries of example simulations are show in Video S9, and modeling of the effect of retrograde flow is in Figure S5.

The main components of the model are summarized in Figure 6A. The cell is modeled as a 2D contour, with 3D measurements of domain area and vesicle volume taken from the corresponding surfaces of revolution. Domain expansion is controlled by a parameter chosen to make the domain first expand and then stall, except where indicated (see Figure S5 for examples). The combined membrane and actin cytoskeleton is modeled as an elastic material with elastic modulus varied via a parameter (k_T_). Dendritic actin polymerization is assumed to occur only at the domain boundary with its force given by the parameter (k_P_). Polymerization creates a reaction force that drives a retrograde flow of F-actin inward, potentially applying a stretching force to the membrane of the cup. We explored its importance by varying how strongly this force is coupled to the membrane of the cup. Actin polymerization within the cup due to formins^48,49^ and the signaling dynamics sustaining PIP3 domains are not considered.

In the first phase of its life (Figure 6A), the PIP3 domain expands producing an F-actin wave at its perimeter that captures more membrane into the cup.^34^ The zone of actin polymerization moves outward and does not remain under the same area of membrane for long, so that the cup remains shallow. As the domain stalls, actin continues to polymerize under the same ring of membrane but does not capture more membrane.

We first consider the case where the force from retrograde flow is not transmitted to the membrane of the cup. Even so, the model readily produces lip closure at different sizes; an elongated cup giving a basal closure when local delamination occurs, as well as a persistent, unclosed cup (Figure 6B). These outcomes are largely controlled by the polymerization force (k_P_; Figure 6C), which drives protrusion of the lip, determining its relationship to the domain boundary. Lip closure occurs if the zone of actin polymerization slips into the cup; closure is prevented if it remains near the lip, pointing outward. This is achieved when the tension force controlled by the elastic modulus (k_T_) constricts the cup, elongating the PIP3 domain and pushing its boundary toward the lip, keeping pace with the protruding lip and maintaining the position of the polymerization zone. Tubes form when parameter values lie just outside the range giving lip closure, whereas values just within this range provides the largest vesicles for a given membrane tension, although these cups take longer to close (Figure 6C; Video S9).

When retrograde flow is allowed to apply force to the membrane, lip closure is faster but not guaranteed (Figures 6D and S5; Video S9). In cases of high membrane tension, the inward force drives the cup deeper into the cell, as is indeed observed in many basal closures (for example, see Video S5).

In narrow tubes produced by the model such as Figure 6Biii, the tension force in a 3D surface of rotation is substantially larger than in the 2D contour due to the high mean curvature of the surface. By adapting the tension force to account for the full 3D surface, we can additionally model basal closure (Figure 6F).

Our modeling produces similar outcomes to that of Saito and Sawai,^50^ which likewise produces lip closure and multiple closures via “closure at the waist.” Their reduced model uses a similar formulation to ours with similar assumptions to 1 and 4 above. One difference is that we treat the coupled membrane and actin cytoskeleton as an elastic material, whereas Saito and Sawai assume membrane tension is modulated by an exocytic process that maintains a constant surface tension. This difference allows tension gradients to form in our model (Figure 6E), which can facilitate tubulation and base closure. Our model does not account for the formation of complex, multi-lobed cups, which may require more complicated modeling of the interaction between signaling dynamics within the PIP3 domain and the 3D geometry of the cup.^50^

Our model therefore reproduces both lip and basal closures, depending on the parameters chosen, showing that the starting propositions provide a route to understanding how macropinocytic cups form and close.

## Discussion

In this paper, we present a model for how macropinocytic cups are shaped and close based on insights gained from lattice light-sheet microscopy and quantitative analysis. We confirm that the major macropinocytic structures in *Dictyostelium* are cups projected from the cell surface, which are built around discrete domains of PIP3. Analysis of these domains reveals underlying principles that lead to a model for how domains both shape macropinocytic cups as they expand and induce closure when they cease expanding.

Macropinocytic cups are shaped by a continuous F-actin scaffold extending from beyond the lip to the base. This is decorated by a distinct pattern of F-actin-binding proteins, consistent with earlier work.^33,43,44^ Based on cryo-electron tomography of the broadly homologous actin waves,^51^ the scaffold is expected to transition from dendritic F-actin near the lip, with many filaments directed against the membrane and therefore able to exert outward force, to less branched filaments parallel to the membrane deeper in the cup. This transition may be produced by processing of dendritic F-actin and the activity of formins that are recruited within cups, activated by Ras and Rac and building F-actin at the base.^48,49^

The plasma membrane must adhere strongly to the scaffold of the cup to prevent delamination in areas of negative curvature, where membrane tension will tend to detach it. Accordingly, cups are enriched in proteins that cross-link membrane and F-actin, including myosin-1^43^ and talin,^52^ whereas the development of membrane tension is shown by the rebound of the membrane from the scaffold when they separate during closure.

This study strongly supports the hypothesis that macropinocytic cups are shaped by a ring of dendritic actin polymerization formed around PIP3 domains.^25^ By tracking the Scar/ WAVE complex (as a proxy for dendritic actin polymerization) throughout the life of macropinocytic cups, we find that it is recruited around PIP3 domains at all stages and whatever the size of the domain. The Arp2/3 complex, which is activated by Scar/WAVE is similarly recruited in rings (Yang et al.^45^; this work) as is vasodilator stimulated phosphoprotein (VASP), which interacts with Scar/WAVE and may accelerate actin polymerization.^53^

When a macropinocytic cup closes, the opposing membranes must be brought together, although they are initially microns apart. A purse-string mechanism, driven by myosin-II contraction at the lip of the cup, has been proposed for phagocytocytic cups.^46,54,55^ Our work provides an alternative mechanism. We find that macropinocytic cups can close at the base, as well as at the lip. Vesicles forming from the base of ruffles have previously been reported in macrophages,^16^ although whether they also undergo delamination remains to be seen.

In our unified model, continued actin polymerization at the lip drives both types of closure: lip closure results when the lip turns inward, and basal closure results when it continues to elongate the cup, stretching the membrane until it delaminates from the scaffold and pinches off a macropinosome.

Although we observe no clear evidence for a role of actomyosin contraction or microtubule motors, we cannot completely dismiss the possibility that they contribute to the inward stretching of the cup into the cell body. Importantly in our model, the force is simply imposed along the membrane and can apply equally to any mechanism. The key point is that any force that pulls the cup base away from the lip helps promote closure under a broader range of biophysical parameter space.

Our modeling treats macropinocytic cups in 2D and only considers mechanical forces. Yet, even within these limitations, it readily reproduces an expanding cup that closes at the lip, and one that does not close at the lip, but elongates, as in base closure. The theoretical model of Saito and Sawai,^50^ although enacted differently, also incorporates the propositions of actin polymerization around PIP3 domains and stalled expansion. It, likewise, reproduces cup formation and closure at the lip and base, indicating that this is a robust consequence of the initial assumptions.

PIP3 domains are complex structures that form spontaneously in the plasma membrane, including at the base of cells.^23,24,35^ They are likely based on a reaction-diffusion system, with auto-catalysis and cross-inhibition of diffusing molecules, including Ras, PIP3, and Rac. PIP3 recruits and activates proteins genetically required for fluid uptake by macropinocytosis, including the protein kinases Akt and serum glucocorticoid regulated kinase 1 (SGK), Leep1 (a regulator of Scar/WAVE), C2GAP2 (a RasGAP), and myosin-1,^45,56,57^ but these are not essential to form PIP3 domains. Active-Ras, which activates PI3-kinase, may be at the core of the domain-forming system, because Ras domains form in the absence of PIP3 domains,^25^ whereas, conversely, PIP3 domains are slaves to Ras activity because they are greatly expanded when Ras activity is increased by deletion of the RasGAPs NF1 or RGBARG.^26,27^

We find that domains produce opposing gradients of PIP3 and PI4,5P2, as also do basal waves.^58^ This is consistent with PI3-kinases producing PIP3 within domains from PI4,5P2, and the PIP3 diffusing out for reversion by PTEN, whereas PI4,5P2 is replenished in the domain by diffusion from the surrounding membrane. This spatial separation of PIP3 production and destruction may be one aspect of the reaction-diffusion system that helps limit domain size. Finally, the ability of PIP3 domains to attract actin polymerization to their periphery may be due to the creation of an annulus, where Rac activation outweighs its inhibition.^27^

Extending our work, we propose that cupped structures of the plasma membrane are more generally created by ringed actin polymerization around PIP3 domains. Mammalian macropinocytic cups also feature a central domain of PIP3, which may similarly organize the cup and guide its closure.^14,59^ Other cupped structures organized by PIP3 domains could include large phagosomes in *Dictyostelium*^19,25,26,60^ and circular dorsal ruffles in mammalian cells.^61^

In summary, we have described macropinocytosis in greater detail than hitherto and gained mechanistic insights, leading to a unified model for how cups close at lip or base. The stalled wave model provides a framework for understanding macropinocytosis both in *Dictyostelium* and more widely.

## Star*Methods

Detailed methods are provided in the online version of this paper and include the following:

- KEY RESOURCES TABLE
- RESOURCE AVAILABILITY
  ⚬ Lead contact
  ⚬ Materials availability
  ⚬ Data and code availability
- EXPERIMENTAL MODEL AND SUBJECT DETAILS
- METHOD DETAILS
  ⚬ *Dictyostelium* transformation
  ⚬ Lattice light-sheet microscopy
  ⚬ Deconvolution and registration
  ⚬ Cell segmentation
  ⚬ Fluorescence projection
  ⚬ Annotation
  ⚬ PIP3 domain segmentation
  ⚬ PIP3 domain grading
  ⚬ Fluorescence measurements
  ⚬ PIP3 domain geometry
  ⚬ 3D maximum intensity projections
  ⚬ Surfaces
  ⚬ Modelling
  ⚬ Base closure
  ⚬ Quantification and statistical analysis

**Table:** Key Resources Table

REAGENT or RESOURCE	SOURCE	IDENTIFIER
**Chemicals, peptides, and recombinant proteins**		
HL5 Medium	Formedium	HLG0103
SM agar	Formedium	SMA0102
Gibco Dialysed fetal calf serum	Thermo fisher	26400044
dihydro-streptomycin	Thermo fisher	15875278
TetraSpeck beads	Thermofisher	T7279
**Experimental models: Organisms/strains**		
*Dictyostelium discoideum* Ax2 (Kay) axenic strain	www.dictybase.org	DBS0235521
Dictyostelium discoideum Ax3 axenic strain	www.dictybase.org	N/A
Dictyostelium discoideum strain DdB	Bloomfield et al.^26^	DBS0350772
Ax2 expressing GFP-PH-PkgE/RFP-lifeact	This work	HM3477 and HM3478
REMI insertion of NAP-GFP into Ax3 NAP null	This work	HM1995
Ax3 co-expressing GFP-NAP1/mCherry-PH-pkgE	This work	HM3485
Ax2 ArpB-GFP knock-in^62^	This work	HM2129
Ax2 ArpB-GFP knock-in/mCherry-PH-pkgE	This work	HM3500
Ax2 co-expressing GFP-Carmill/mCherry-PH-pkgE	This work	HM3498
Ax2 co-expressing GFP-Myo1B/ mCherry-PH-pkgE	This work	HM3499
Ax2 co-expressing GFP-Myo1E/ mCherry-PH-pkgE	This work	HM4002
Ax2 co-expressing GFP-PH-pkgE/mCherry-PTEN	This work	HM4003
Ax2 co-expressing GFP-MhcA/ mCherry-PH-pkgE	This work	HM4013
Ax2 co-expressing GFP-2xPH-TAPP1/mCherry-PH-PkgE	This work	eJSK09
Ax2 co-expressing GFP-Nodulin/mCherry-PH-PkgE	This work	eJSK15
Ax2 co-expressing GFP-P4M/mCherry-PH-PkgE	This work	eJSK18
Ax2 co-expressing GFP-a-tubulin/mCherry-PH-PkgE	This work	eJSK19
Ax2 PI3K1-5 quintuple knockout	Hoeller et al.^38^	HM1200
DdB co-expressing GFP-PH-PkgE/RFP-lifeact	This work	HM3481
Ax2 PTEN knockout	Hoeller et al.^38^	HM1289
Ax2 RGBARG knockout	Buckley et al.^27^	JSK02
**Recombinant DNA**		
GFP-PH-PkgE/RFP-lifeact co-expression vector	This work	pPI304
mCherry-Ph-PkgE expression vector	This work	pPI167
GFP-Carmil/mCherry-PH-PkgE co-expression vector	This work	pPI36
GFP-Coronin/mCherry-PH-PkgE co-expression vector	This work	pPI78
GFP-Myo1B/mCherry-PH-PkgE co-expression vector	This work	pPI32
GFP-Myo1E/mCherry-PH-PkgE co-expression vector	This work	pPI87
GFP-PH-PkgE/mCherry-PTEN co-expression vector	This work	pPI356
GFP-mhcA/mCherry-PH-PkgE co-expression vector	This work	pDM1485
GFP-2xPH-TAPP1/mCherry-PH-PkgE co-expression vector	This work	pJSK696
GFP-Nodulin/mCherry-PH-PkgE co-expression vector	This work	pJSK706
GFP-P4M/mCherry-PH-PkgE co-expression vector	This work	pJSK708
GFP-a-tubulin/mCherry-PH-PkgE co-expression vector	This work	pJSK709
**Software and algorithms**		
Matlab 2021b	Mathworks	https://uk.mathworks.com/products/matlab.html
Curvature Enhanced Random Walker	Lutton et al.^30^	https://pilip.lnx.warwick.ac.uk/TMI_2020/
MiCellAnnGELo	Platt et al.^63^	https://github.com/CeNDynamics/MiCellAnnGELo
Matlab image and surface analysis tools	This paper	https://wrap.warwick.ac.uk/175423
Mathematical model of macropinocytosis	This paper	https://wrap.warwick.ac.uk/175423
Fiji scripts for image conversion and generating 3-way projections	This paper	https://wrap.warwick.ac.uk/175423
PIP3 Domain grading interface	This paper	https://wrap.warwick.ac.uk/175423

## Resource Availability

### Lead contact

Further information and requests for resources and reagents should be directed to and will be fulfilled by the lead contact, Jason King (jason.king@sheffield.ac.uk).

### Materials availability

Plasmids generated by this study are available though Addgene or request from the lead contact.

### Data and code availability

- Raw imaging data is freely available to share upon request.
- All original code is deposited in the Warwick Research Archive Portal (WRAP) under the persistent URL: https://wrap.warwick.ac.uk/175423.
- Any additional information required to reanalyze the data reported in this paper is available from the lead contact upon request.
- Requests for information on image processing and computation should be directed to Till Bretschneider (till.bretschneider@warwick.ac.uk).

## Experimental Model and Subject Details

All *Dictyostelium discoideum* strains used in this study derive from the axenic laboratory strain Ax2(Kay),^26^ with the exception of the non-axenic isolate Ddb. Reporter strains are listed in key resources table and derive from Ax2(Kay)^26^ unless otherwise indicated. They were grown at 22°C either in HL5 axenic medium (Formedium, Hunstanton, UK) in shaken suspension at 180 rpm, or on tissue-culture plates; or in association with *Klebsiella aerogenes* on SM agar plates. Non-axenic or poorly growing axenic strains, such as DdB, were grown in HL5 reinforced with 10% dialysed FCS (Gibco).

Cells for microscopy were grown for several days in HL5 medium to maximally up-regulate macropinocytosis. They were prepared for microscopy by incubation for 2-24 hr at approximately 10^5^ cells/cm^2^ in 6-well tissue culture dishes in SUM low fluorescent medium (20 mM KH_2_PO_4_, 4 mM arginine, 3.7 mM glutamic acid, 8.5 mM lysine, 55 mM glucose, 2 mM MgSO_4_, 0.1 mM CaCl_2_ brought to pH 6.5 with H_3_PO_4_, plus 100 mg/ml dihydro-streptomycin (Gibco).^31^

## Method Details

### *Dictyostelium* transformation

Cells were transformed with plasmids from the Paschke/Veltman series,^64,65^ generally to express two reporters, one of which was for PIP3 (PH-PkgE^25^). The PI45P2 reporter used was Nodulin,^62^ PI34P2 was PH-TAPP1^24^ and PI4P was P4M.^66^ Microtubules were imaged using GFP-a-tubulin^67^ and F-actin by LifeAct.^32^ Cells were transformed by electroporation and selected on pre-grown bacteria with the appropriate drug.^64^ Plasmids are available from Addgene, or from the authors by request.

### Lattice light-sheet microscopy

LLSM was carried out using a 3i Lattice Light Sheet instrument with a 0.71 NA long-working-distance water-immersion excitation objective and a 1.1 NA water immersion emission objective with a total magnification of 62.5x. Images were captured on two Hamamatsu ORCA-Flash 4.0 v3 sCMOS cameras (single channel each). The sheet pattern was a Bessel lattice of 50 beams, with an inner and outer numerical aperture of 0.493 and 0.550 respectively. The thermoelectric cooler system was set to 17°C to achieve a temperature of 22°C in the sample chamber.

Before imaging, 10 ml of TetraSpeck beads (Thermofisher) were dried to a 5 mm coverslip (Mentzel Glasser #1, VWR) and mounted on the sample holder using vacuum grease (Dow Corning high vacuum grease, VWR). 10 ml of medium (SUM) was added to the bath and the sample left to equilibrate in the microscope for 30 min before being used for alignment and PSF measurement. Cameras were aligned by exciting the beads at both 488 nm and 560 nm and a PSF measurement taken for localization.

*Dictyostelium* cells in 6-well plates were detached by pipetting up and down and allowed to settle for at least 15 min onto 5 mm coverslips added to the wells. A coverslip was mounted on the sample holder and transferred to the microscope containing 10 ml SUM in the bath. Samples were allowed to equilibrate before recording. Images were recorded in two channels using 1-2% and 5-10% laser powers for 488 nm and 560 nm respectively, depending on the reporter. 3D volumes were recorded at 0.3 μm step size (0.163 μm deskewed) for 120-150 planes with a 5-10 ms exposure. This resulted in an overall speed of 2.24-3.12 seconds per volume, depending on the configuration, and an (x, y)-resolution of 0.104 μm/pixel. Volumes were recorded at this rate for 3–10 min. All Videos and images are maximum intensity projections.

### Deconvolution and registration

Because the same microscope was used for all experiments, deconvolution was made more efficient by condensing one PSF for each channel and applying it to all videos for deconvolution. Comparisons between these deconvolved images and a sample of videos deconvolved using PSFs generated immediately prior to the videos showed no substantial change in intensity but did yield a small translation of the image. This translation was accounted for by measuring cross-correlations of the channels within a range of 5 voxels (twice the largest observed translation) and selecting the translation that yielded the maximum average cross-correlation for the whole video. Deconvolution was performed using the Richardson-Lucy method^68^ (50 iterations).

### Cell segmentation

All videos were segmented using the curvature-enhanced random walker.^30^ The segmentation method itself requires only two parameters. However, some pre- and postprocessing is often required to enable accurate segmentation, which increases the number of parameters.

To identify parameter configurations for the segmentation, videos assigned one of three groups: (1) those with PIP3 in the red channel, (2) those with PIP3 in the green channel without LifeAct, and (3) those with PIP3 and LifeAct. A parameter configuration that yielded accurate segmentation for most videos in each group was identified. Segmentations were manually verified, and any errors to segmentation of PIP3 patches were excluded from subsequent analysis. Parameter configurations for videos with a low segmentation accuracy of PIP3 patches were adjusted to improve the accuracy.

Videos in group (1), which contains the majority of the videos, were segmented using similar parameter configurations, with 70% using a single configuration. Similarly, group (2) mostly used the same parameter configuration (80%), with small variation from this configuration otherwise. Group (3) had the highest variation in parameter configurations, due to the fact that neither PIP3 nor actin have a strong presence in the cytoplasm leading to segmentation errors. However, there was a large overlap in parameter configurations, with at most four distinct values for any given parameter.

The segmentation error rate for each PIP3 domain was calculated by manually grading the segmented surfaces (further details below). An average of 6.97% +/- 0.83% (standard error) of frames were identified as having a segmentation error and were excluded from subsequent analysis.

### Fluorescence projection

Triangulated surface meshes were obtained from the segmented videos using Matlab’s isosurface algorithm. These meshes take the form of a set of vertices and a set of triangular faces that connect the vertices. In order to measure the fluorescence on the membrane, the fluorescence values in the image were projected onto the surface. This was performed using line scans along the direction normal to the surface. For a given vertex *v* on the surface, the image voxel coordinates along a line *l* normal to the surface through *v* were calculated. Line scans were truncated to avoid including voxels containing membrane at another part of the cell surface; if a point *p* in *l* is closer to another point on the surface than to *v*,then *l* is truncated at *p* and any points beyond *p* are discarded from *l*. A minimum distance of 2 voxels (approx. 0.2 μm) was imposed on the truncation to prevent zero-length line scan in highly curved parts of the surface. Lines had a maximum length of 1 μm (1.5 μm for actin fluorescence). Each vertex was assigned the maximum fluorescence value along its line scan. Background subtraction of the fluorescence was applied to the image prior to projection using a top hat filter of radius 10 voxels for all images.

For the purpose of computing line scans, all surfaces were smoothed using Laplacian smoothing. Projecting actin fluorescence onto the surface additionally required shrinking the surface areas of high mean curvature. This is because the actin cortex lies slightly below the membrane, and therefore the peak fluorescence is farther from the surface than other markers. Given that highly curved areas introduce extreme truncation of line scans, this would lead to the actin at the lip of the macropinocytic cups being reduced. We solved this problem by shrinking the surface under mean curvature flow, which forces the surface to shrink in highly curved regions. In order to avoid simultaneously shrinking the cups during this process, mean curvature flow was only applied in regions of positive mean curvature. Positive mean curvature flow was applied until any point on the surface had moved up to a maximum of 5 μm from its original position.

### Annotation

Surfaces were annotated using MiCellAnnGELo, a VR software for rapid annotation of surfaces.^63^ Markers were placed on the surface using the marker placement tool in MiCellAnnGELo at locations within PIP3 domains in each video. For *de novo* domain formation events, markers were placed in frames prior to this event at the approximate location of the forming domain. Similarly, after domain elimination events markers were placed in subsequent frames at the approximate location of the extinguished domain. When a single domain contained more than one cup, separate markers were placed in each cup. Markers were grouped in sequences by first measuring the distance from markers in each frame to markers in the following frame and pairing each marker to the closest marker in the following frame. This pairing process was repeated in reverse, and two markers were grouped together if they paired in both directions. Occasionally, markers moved large distances between frames, for example during a closure event, leading to them no longer pairing with the corresponding marker in the previous frame. These markers we regrouped later in the pipeline by comparing manual annotations of sequences and joining sequences with no overlapping frames, but with the same manual annotations in each frame.

### PIP3 domain segmentation

Domains were identified around each marker using Otsu thresholding^37^ of the PIP3 fluorescence values on the surface. Threshold values were computed for each marker using the histogram from vertices nearest to the given marker in the geodesic sense. In cases where structures such as cup lips or ruffles present as thin protrusions with PIP3 on only one side of the structure, a low level of PIP3 will be projected onto the other side in the surface representation due to spreading of the fluorescence during image capture. In order to avoid including the opposite side of such structures in the domain, mean curvature is used to separate the two sides, while also providing geometric definitions for *cups* and *lips*.

For a given marker, a *cup* is defined as a connected area on the surface about the marker, where all vertices have PIP3 intensity above the Otsu threshold and mean curvature less than 0 (concave). The lip is defined as the band of vertices, grouped by geodesic distance from the cup boundary, with the highest average mean curvature (highly convex). The *domain* about the given marker is the set of all vertices within the lip contour with PIP3 value above the Otsu threshold.

In cases where *cups, domains*,or *lips* for two markers intersect, it is necessary to further segment these structures to maintain correspondence with the markers. This segmentation was performed by dividing the structures along the line of equal geodesic distance between the two markers. *Domains* that required segmentation in this manner were automatically graded as being split *domains*.

For cases where the PIP3 intensity was below the threshold value at the marker, the marker vertex and all vertices sharing a face with that vertex were marked as the *domain* for ease of processing. The frame when this false *domain* changes to a true *domain* were automatically marked as *domain* formation events, and the reverse as *domain* elimination events.

Each of the *cups*, *domains*, and *lips* were additionally smoothed by dilating (adding neighboring vertices, applied twice), filling (adding connected components surrounded by the vertices of the given feature in the mesh graph), and eroding (the reverse of the dilation operation).

### PIP3 domain grading

Each domain was subsequently graded manually for each frame to mark the events of *De novo* domain formation, domain elimination, lip closure, base closure, failed closure, and domain splitting. Domains were also assessed for cell segmentation and domain segmentation errors, which were noted and excluded from subsequent analysis. The automated grading above provided an initial set of gradings, which were adjusted where necessary.

Gradings were made by visual comparison of the surface and the 3D maximum intensity projection of the video. A custom Jupyter notebook was designed to allow rapid grading of domains, using ITKWidgets with PyVista to render the surfaces.^69^ This notebook allows the user to change surface colours between fluorescence and domains, as well as providing controls for opacity and colour scaling. Buttons for grading allow the marking of base, lip, and failed closures, as well as domain splitting, segmentation and domain detection errors, and noting the start and end of the domain.

For a given marker, frames between domain start and end gradings are taken to be the times when the domain is present. For domains with no start frame (either formed prior to the start of the video or formed from splitting), the domain is assumed to start from the first frame that the corresponding marker is present. Similarly, domains with no end frame are assumed to end when the marker is no longer present. Frames where the domain was not automatically detected for a given marker (see previous section) but manually marked as having a domain (and vice versa) were automatically graded as having a domain segmentation error.

In the subsequent analysis, measurements of a domain graded as split or to have an error in a given frame were excluded for that frame. Where time-dependent measurements were taken, domains which were graded as split at any time in the interval being measured were excluded.

### Fluorescence measurements

Fluorescence measurements were made relative to the domain boundary. Additionally, the mean curvature of the surface was computed and processed in this manner, without the background subtraction step. The surface outside and inside this boundary was partitioned by geodesic distance to the boundary, where bands outside the domain were equally spaced at 0.2 μm, and bands inside the domains were equally spaced to 0.1*domain depth (defined below). Using a normalized distance inside the domain allows comparison between domains of different sizes. The average fluorescence values were taken in each band, giving a 1-dimensional profile of the fluorescence values for each time point. For cells with multiple domains, bands around a domain were restricted to points on the surface closest to the given domain’s boundary in the geodesic sense.

For each domain, the background fluorescence measurement in each frame was taken to be the average fluorescence 5 μm outside the domain. The median background fluorescence over the lifetime of the domain was taken to be the overall background. Fluorescence values were normalized by dividing by this background value (fold normalization).

To give an overall profile of fluorescence and curvature, the average normalized 1-dimensional profile was taken for each domain, and these averages aggregated to give the plots shown in Figure 2.

For time-dependent analysis, closure events were taken to be the temporal reference points. The number of domain sequences was reduced to include only those that contained the closure event being investigated (either lip or base closure), and the required time interval preceding the event. Due to variable time steps of the videos and some frames being rejected in the quality control step above, fluorescence values were linearly interpolated at every 2.5 seconds prior to the closure time. The average interpolated 1-dimensional fluorescence profiles for each time point were thus computed and plotted as colour values on a 2D image. The image was distorted to account for distance normalization of the profiles by scaling the part of the image representing points inside the domain by the average domain depth along the distance axis.

### PIP3 domain geometry

Geometric measurements for each domain were made using the surface geometry: the perimeter was taken to be the total length of edges along the boundary of the domain; the area was given by the total area of all faces in the domain, and the depth was taken to be the maximum distance of any point in the domain to the domain boundary.

In order to compare the geometry of multiple domains, two approaches were used. In the first, all domains captured undergoing *de novo* formation, lip closure, and elimination were aggregated by normalizing in both time and the geometric measurement, allowing the observation of general trends in the geometry (Figure 5). Time normalization was achieved by setting the formation time to 0 and closure time to 1 and interpolating values linearly at 20 evenly spaced time points between these two events. Measurements were normalized by dividing by the 75^th^ percentile. In the second comparison, measurements for the last minute prior to closure (Figure S4) were aggregated using the closure time as a reference point. Due to the variable time between frames and some frames being excluded during quality control, values were linearly interpolated to obtain a series of values uniformly spaced in time for each data-set, which could then be aggregated at each time point. An interval time of 2.5 s was taken, since this is close to the frame interval in most of the videos. The reference time for each event was calculated as halfway between the frame containing the event and the previous frame.

### 3D maximum intensity projections

It is necessary in the above to visually compare 3D surface data with 3D volume data. To enable rapid visual inspection of cells, 3-way maximum intensity projections were generated for each frame by taking the projections in each of x, y, and z after rescaling in z to produce isotropic resolution. These images were combined into one as a montage, placing the projection in z (represented in the (x, y)-plane) in the top left, the projection in y ((x, z)-plane) in the bottom left, and the projection in x ((y, z)-plane) in the top right. This yielded a view where the projections with lower resolution (x and y) could be easily compared to the high-resolution projection (z) due to the alignment of the common axes. These projections allowed easier identification of closure events than the projection in z alone because they provide some of the 3D information lacking in the single projection.

### Surfaces

Surface rendering for purely visualization purposes was performed in Matlab (see above for annotation using VR and grading using ITKWidgets in Jupyter). The surfaces were preprocessed by applying Laplacian smoothing (3 iterations), to provide a smoother surface to view. A customized Matlab live script was used to allow fast rendering of the surfaces. Surface colour and opacity can be linked independently to fluorescence, curvature, or PIP3 patches. These values can be scaled to allow better visualization of the surface for the length of the video.

### Modelling

A mathematical model is used to simulate the behaviour of cells during macropinocytosis. Our aim with this model is to show the feasibility of cup formation and closure resulting from a polymerization force acting at the boundary of a PIP3 domain, given that the domain area expansion stalls at some time point.

The model is based on a previous contour model used to study blebbing.^70^ The total force, F_T_, at a given vertex is

$${\text{F}}_{\text{r}}={\text{F}}_{\text{tension}}+{\text{F}}_{\text{bending}}+{\text{F}}_{\text{area}}+{\text{F}}_{\text{poly}},$$

where F_tension_ is the force due to membrane tension, modelled as elastic stress induced by stretching the contour, F_bending_ is the force due to bending of the membrane, F_area_ is a force preserving the area inside the contour, and F_poly_ is the normal force due to actin polymerization. The first two of these forces are calculated in the same manner as previously,^70^ F_area_=k_A_(A/A_0_-1) for area inside the contour A with initial value A_0_ and force coefficient k_A_, and F_poly_ is applied along the normal direction at the domain boundary as described below. Bending and area force coefficients are kept constant in all simulations.

While a contour model is 2D in nature, we may interpret it as approximating a slice through a cell with rotational symmetry, allowing us to calculate values for the perimeter (Figure 6D) from 2D measurements.

We define a PIP3 domain on the contour as a portion of the contour with length d, expanding at a rate v_P_/d for a constant value v_P_. This gives a slowing rate of expansion in length, while the domain area, calculated using a surface of rotation, stops expanding when approaching lip closure due to the shape changes of the domain.

The contour is initiated as a circle with radius 5 μm, discretized using 1000 line segments, and PIP3 patch of initial width 0.02 μm. Throughout the simulation, the force F_poly_ is applied as a constant multiple, k_P_, of a Gaussian function centered at the domain boundary on both sides with standard deviation 0.25 μm. A domain expansion speed of 0.1 / d μm s^-1^ for domain width d is used for all experiments.

### Base closure

This 2D model can produce range of behaviors of cups (Figure 6B). If we also consider the 3D membrane tension force within a cup (Figure 6E) and assume a rigid actin scaffold is holding the membrane in place, we can identify a potential mechanism for base closure, given a partial delamination of the membrane from the actin scaffold (Figure 6F).

If a portion of the membrane in the cup, approximating a cylinder of length l and width w, delaminates from the actin cytoskeleton, then it will start to move under membrane tension. Here, we model the tension force as proportional to mean curvature, while assuming all other forces are negligible. If the aspect ratio l/w < 0.66 (approx.), then the membrane in the cylinder can attain a minimum surface area without closure. We therefore enforce an aspect ratio greater than 0.66^71^ when setting the length of the delamination zone, allowing the membrane to continue to move inwards until closure. It is conceivable that smaller initial delamination lengths could also lead to base closure, since tension at the edges of the delaminated region would be increased, leading to further detachment from the actin cytoskeleton. A similar effect can also occur during lip closure, but is not essential for closure in this case.

### Quantification and statistical analysis

Statistical results are presented in Figures 2, 3, 5, and S4, and Table 1. In all cases, n is given either in the figure/table or the legend, along with clarification of whether this refers to the number of videos or cups. Descriptions of statistics presented in line graphs are given in the figure legends. Details of fluorescence and geometry measurements were computed and how the space/time plots were generated are given in the measurements section of the methods. Details of how cups were selected and graded are given in the PIP3 domain grading section of the methods. All quantification was performed in Matlab.

## Supplementary Material

Figs S1-S5

Vid S1

Vid S2

Vid S3

Vid S4

Vid S5

Vid S6

Vid S7

Vid S8

Vid S9

## Figures and Tables

**Table 1:** Measurements of macropinocytic cup formation^a^

Measurement strain	Mean ± SD	Videos (n)	Time total (min)	Domains measured (n)
**Ax2 strain**				
Domains per cell	2.71 ±1.72	54	225	–
MP formation per cell (min^−1^)	1.80 ± 1.20	54	225	–
Lip closure per cell (min^−1^)	1.23 ±0.88	54	225	–
Base closure per cell (min^−1^)	0.56 ± 0.56	54	225	–
Domain expansion rate^b^ (μm^2^ s^−1^)	0.30 ± 0.34	27	64.7	112
Domain maximum area^b^ (μm^2^)	27.2 ± 14.9	44	146	256
Cup maximum depth^b^ (μm)	2.69 ± 1.07	44	146	256
Domain maximum domain^b^ (μm)	27.5 ± 10.5	44	146	256
Domain expansion rate^c^ (μm^2^ s^−1^)	0.35 ±0.16	11	17.2	13
Domain maximum area^c^ (μm^2^)	29.5 ± 9.43	11	17.2	13
Cup maximum depth^c^ (μm)	2.88 ± 0.60	11	17.2	13
Domain maximum perimeter^c^ (μm)	28.0 ± 7.72	11	17.2	13
**Other strains**				
MP formation per cell (min^−1^) DdB (NF1+)	2.52 ±1.47	2	12.9	–
MP formation per cell (min^−1^) HM1200 (PI3K1-5-)	0	5	21.1	–
MP formation per cell (min^−1^) HM1289 (PTEN-)	0.58 ± 0.07	3	15.7	–
MP formation per cell (min^−1^) RGBARG-	2.36 ± 0.61	3	21.2	–

a Domains were identified computationally as described in the STAR Methods. Data were extracted from the stated number of videos, total elapsed time, and domains. Expansion rate and geometric measurements were obtained from a set of single domains, which did not split in the observation window. Parameters were obtained either from the set of domains used in Figure 5, which could be followed from start to finish (salmon) or larger sets, which could be followed for only the start or only the finish of their life (green). Domain expansion rate was calculated for the first 30 s after *de novo* domain formation as the gradient of the change in area, calculated using a line of best fit.

b Single cups, not complex ones.

c Single cups used for Figure 6.

## Highlights

- Macropinocytic cups can close by two different mechanisms—at the lip or base
- Lip closure is initiated by stalled expansion of the PIP3 domain
- Base closure is driven by membrane tension and delamination
