# Supplementary material for: Formation and closure of macropinocytic cups in Dictyostelium
Source: Curr Biol. Author manuscript; Available in PMC 2023 Aug 18. (PMC7614961; doi:10.1016/j.cub.2023.06.017)
Supplement: Figs S1-S5 [file EMS184995-supplement-Figs_S1_S5.pdf]

Current Biology, Volume *33*

Supplemental Information

Formation and closure of macropinocytic cups in *Dictyostelium*

Judith E. Lutton, Helena L.E. Coker, Peggy Paschke, Christopher J. Munn, Jason S. King, Till Bretschneider, and Robert R. Kay

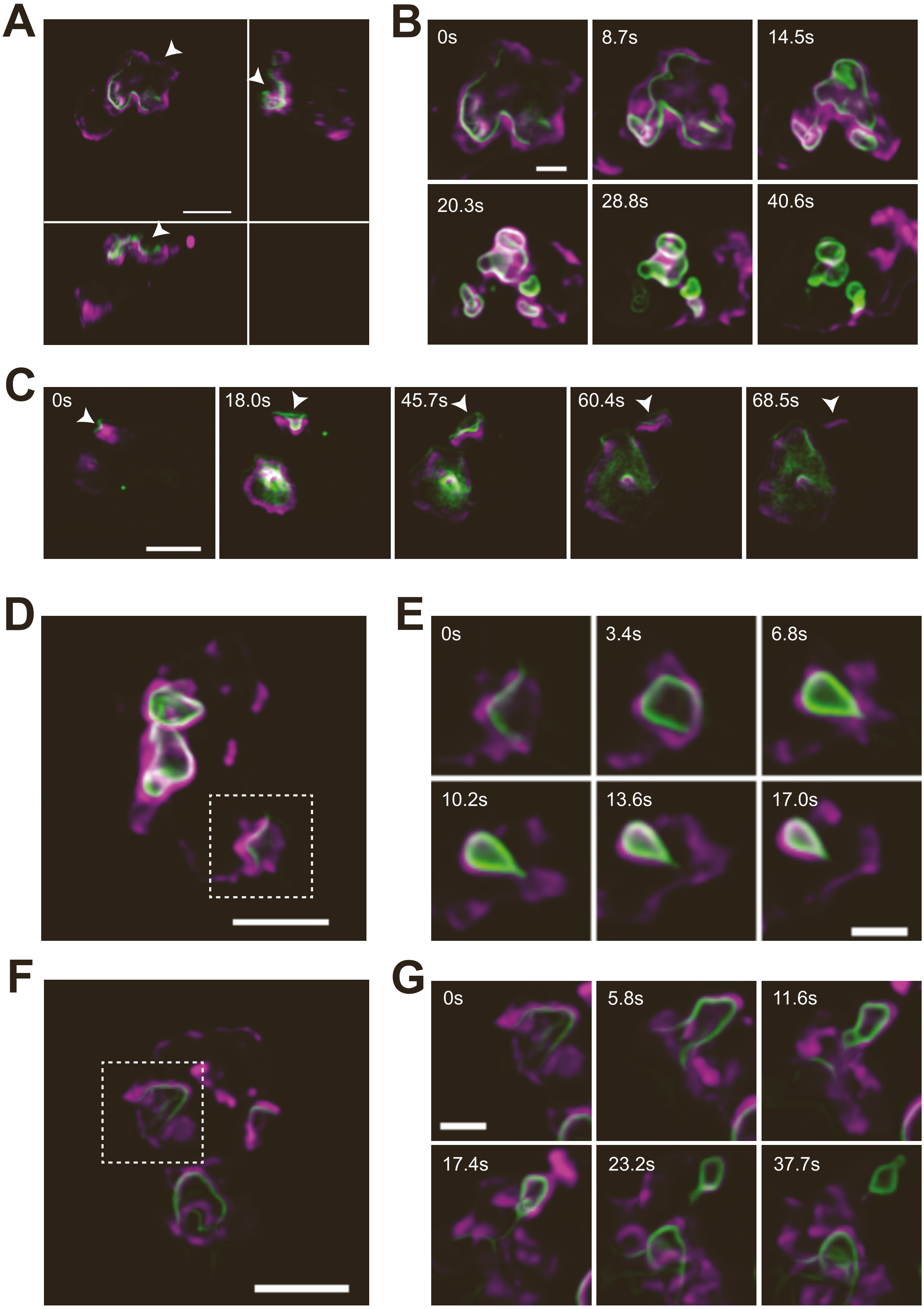

Figure S1: Complex, failed and non-axenic macropinocytosis, related to Figure 1. (A, B) Closure of a complex, multi-lobed cup produces several macropinosomes almost simultaneously: (A) orthogonal views of a single Ax2 cell, the macropinocytic region of which is followed in (B). (C) Macropinocytic cup failure (arrowed) in Ax2, where after a failed attempt at closure (18.0 sec) the PIP3 domain fades away, followed by the F-actin. (D-G) Macropinocytosis is similar in non-axenic DdB cells, which have a functional NF1 RasGAP, to axenic Ax2 cells where NF1 is deleted. (D, E) closure at the lip; (F, G) closure at the base. As in Ax2 cells, DdB cells form PIP3 domains that mark macropinocytic cups, and these cups can close at lip or base. Transient tethers between the newly closed macropinosome and the cell surface also frequently form. The essential features of macropinocytosis are therefore very similar to Ax2 cells. All cells express the PIP3/lifeAct reporter combination. DdB cells were grown in HL5 supplemented with 10% FCS to stimulate growth and macropinocytosis. Scale bars B,C,D,F = 5 µm; E,G = 2 µm. See Movie 4 for full timelapses.

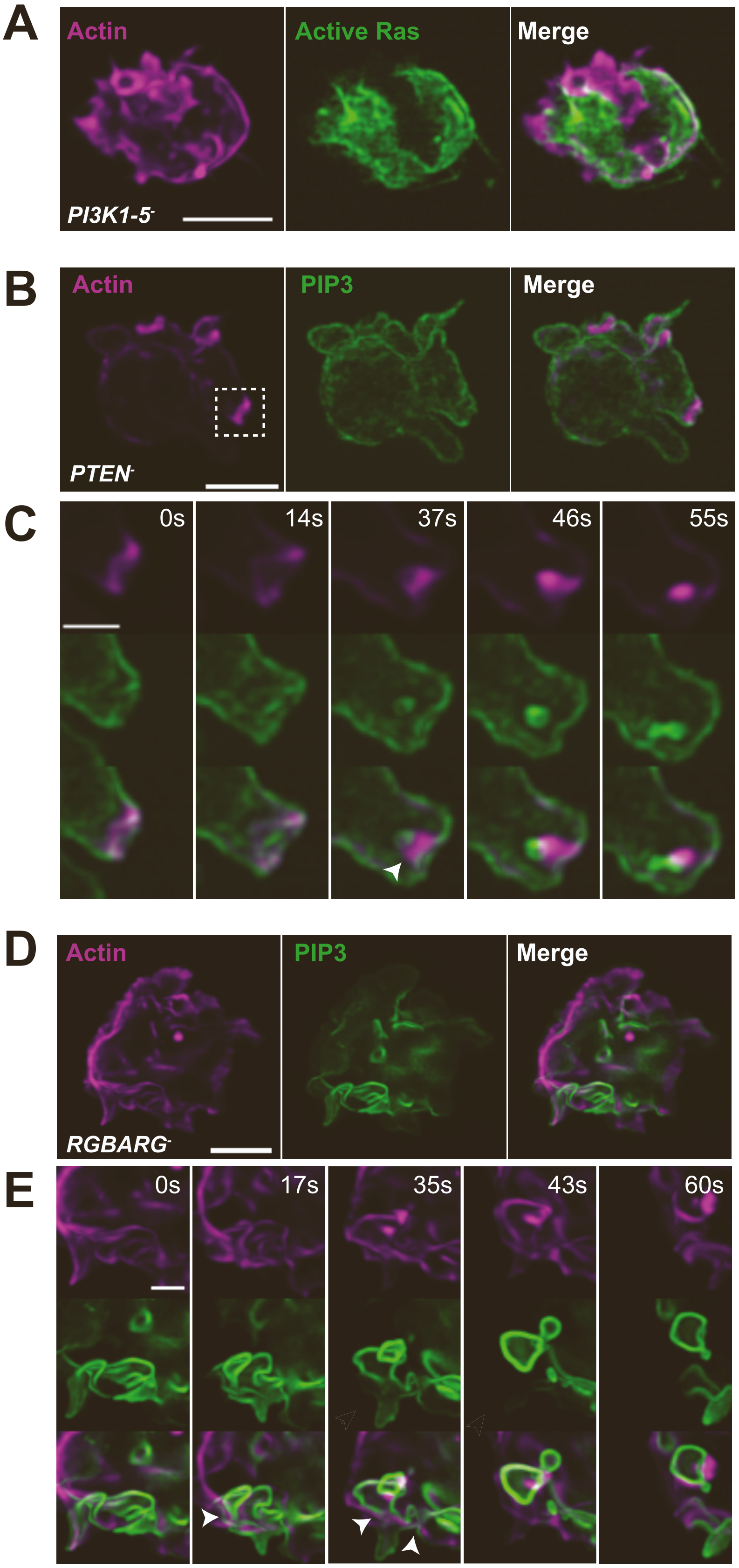

Figure S2: Mutants in Ras/PIP3 signalling are defective in macropinocytosis, Related to Figure 1. (A) PI3-kinase mutant (HM1200): this mutant lacks all 5 Ras-activated PI3-kinases present in the genome and has about 10% of the PIP3 and takes up less than 10% of the fluid of its parent. It still makes domains of active Ras but no macropinosomes were observed. (B, C) PTEN- mutant (HM1289): this lacks PTEN, which reverts PIP3 to PI4,5P2 and in consequence has greatly elevated PIP3 levels (~10-fold) giving elevated PIP3 across the plasm membrane. It produces occasional, small macropinosomes as shown here. (D, E) RGBARG- mutant: this lacks a RasGAP and in consequence makes enlarged PIP3 domains, which are inefficient at macropinocytosis. Numerous small macropinosomes are formed by base closure. Representative images of each mutant expressing paired reporters for PIP3, or in the case of PI3K1-5- for active Ras (RBD - Ras Binding Domain) paired with lifeAct for F-actin. Rates of macropinosome formation are given in Table S1. Fluid uptake rates and PIP3 levels (HM1200 and HM1289) have been determined previously 19,22,25,27.
Scale bar = 5µm.

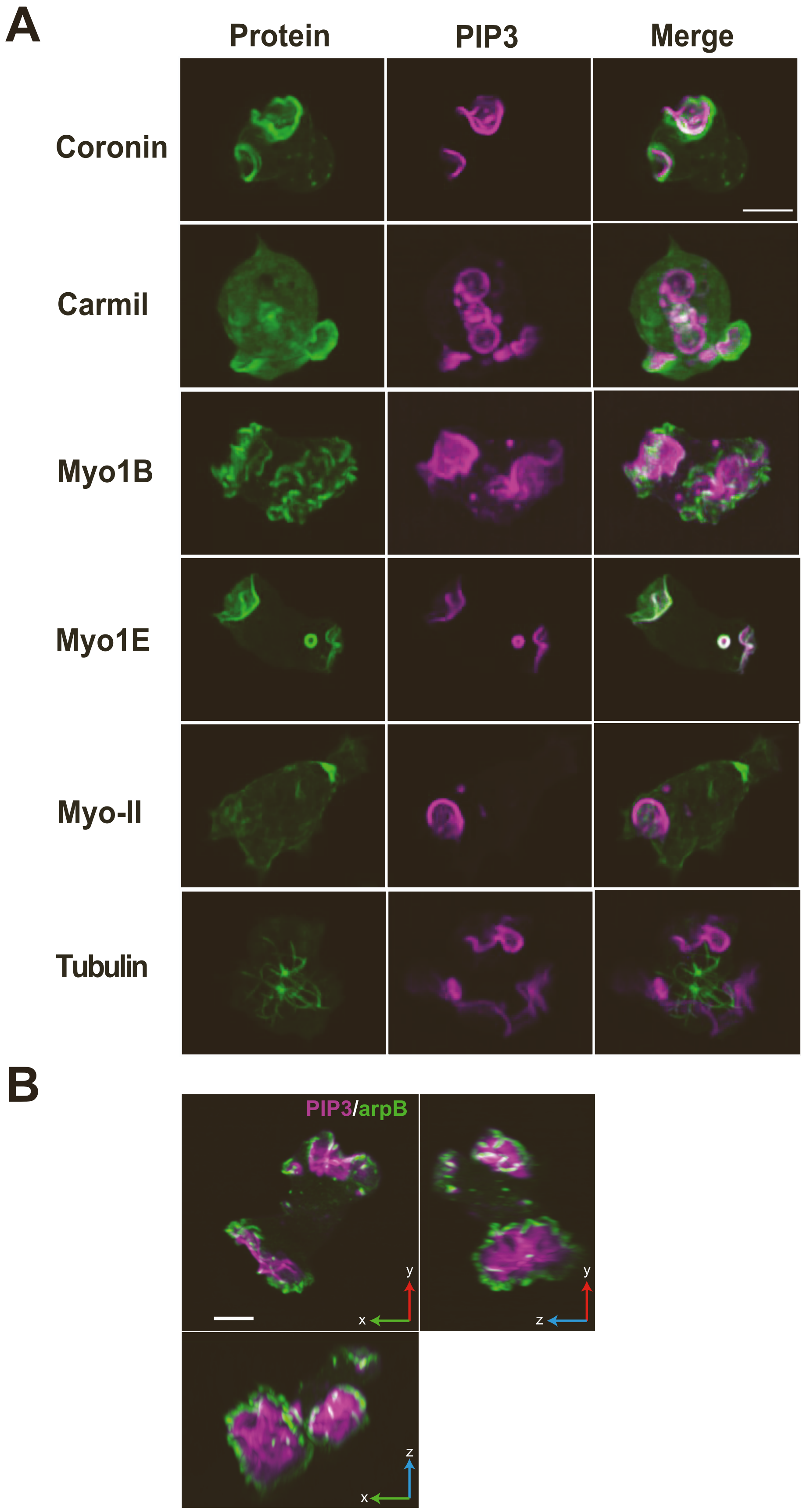

Figure S3: Distribution of cytoskeletal reporters, Related to Figure 2. (A) Representative images showing GFP fusion reporters (green) for the selected cytoskeletal protein with a PIP3 reporter (magenta). Coronin, carmil, myosin1B (Myo1B) and myosin1E (Myo1E) are all concentrated in macropinocytic cups, as previously described, with the PIP3-binding Myo1E closely matching the PIP3 distribution. Myosin-II (Myo-II) is not detected in macropinocytic cups at any stage but can accumulate at the contractile rear of the cell. Microtubules, marked by GFP-tubulin, move freely in the cytoplasm, and sometimes appear to touch macropinocytic cups, but no stable association is detected. Ax2 cells expressing the reporters listed in Key resources table. (B) Maximum intensity and orthogonal projections of Ax2 cells co-expressing GFP-ArpB, a reporter for the ARP2/3 complex and PIP3 (magenta). Scale bars = 5µm.

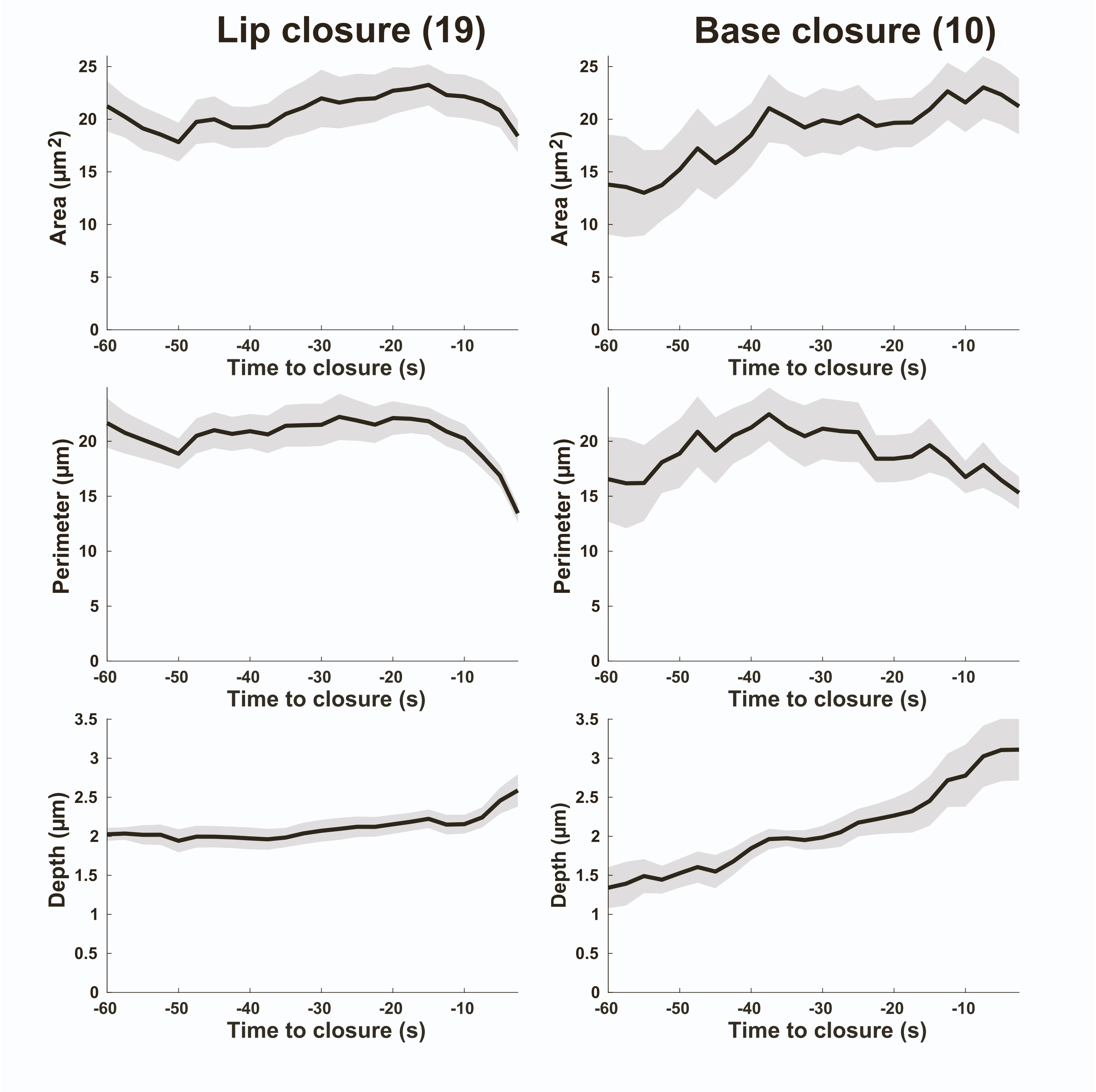

Figure S4: The last minute before closure of cups at lip or base, related to Figure 5. Macropinocytic cups that could be followed for the last minute before closure were classified as either closing at the lip (n=19) or base (n=10) and the geometric parameters of the PIP3 domain computationally extracted as described in the Methods. Ax2 cells expressing the PIP3/lifeAct reporter combination. The time of closure (0 sec) is taken as the last frame in which the macropinocytic cup is unclosed.

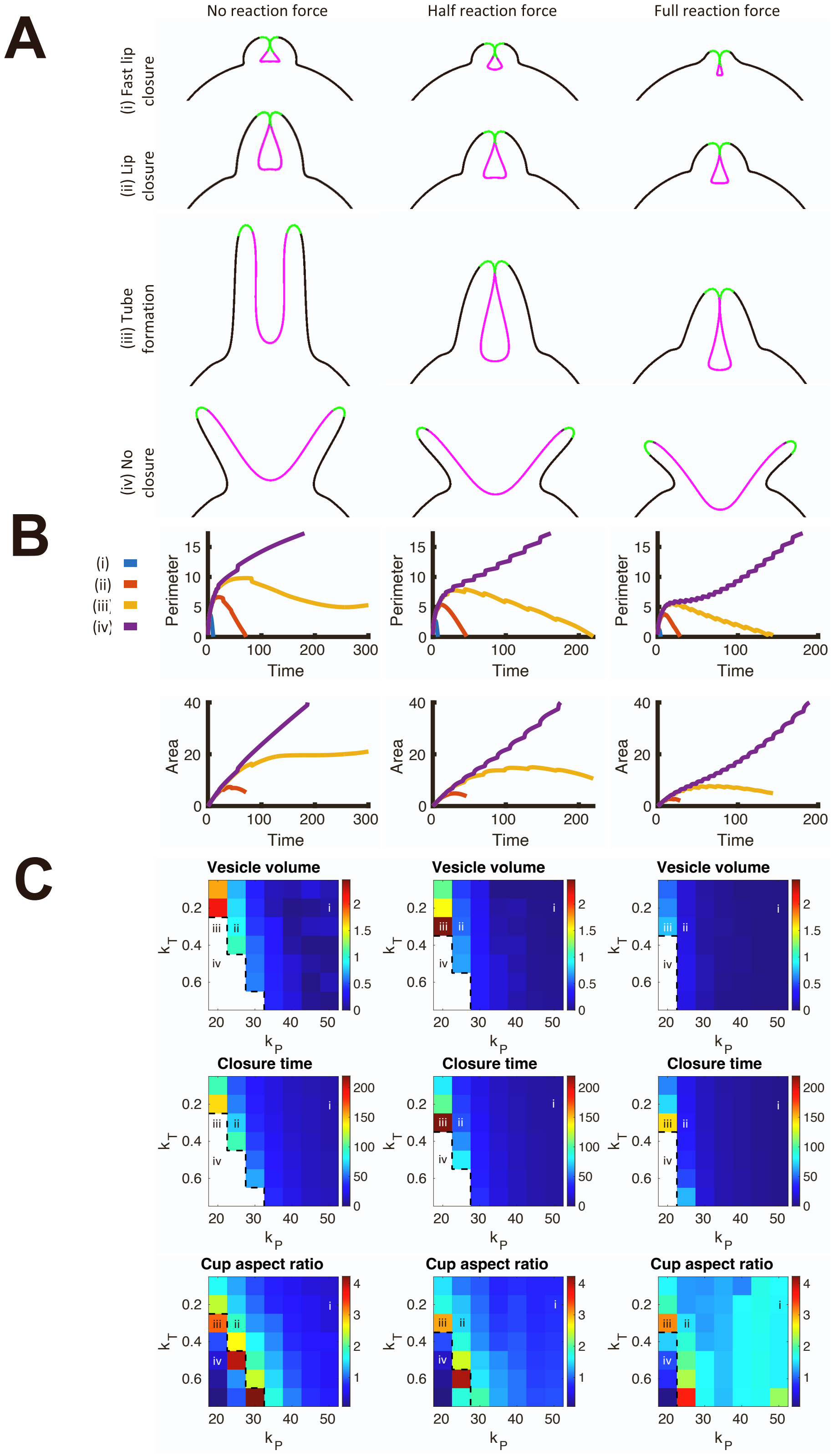

Figure S5: Additional model results, related to Figure 6. The reaction force created by actin polymerization under the membrane can be transmitted to the membrane of the cup by retrograde flow of F-actin from the polymerization site and hence influence the closure process. (A) The effect of different transmission efficiencies was modelled: 0%(left), 50% (middle), and 100% (right). (i)-(iv) show outcomes of the model using the same values of kT and kP as the corresponding panels in Figure 6B. (B) Perimeter plots for each of (i)-(iv) show the impact of transmitting the force from retrograde flow on the lip, while area plots show that the area stalls in all cases where the lip moves inward. (C) Phase plots show that increasing the transmis- sion efficiency increases the chance of closure (shrinking of white region in top two phase plots), while reducing time to closure and vesicle volume. It also increases the aspect ratio of the cup in general, but the boundary between lip and failed closure, where aspect ratio is highest, changes for different levels of trans- mission, leading to some values decreasing with increased transmission.
